# Population structure analysis using rare and common functional variants

**DOI:** 10.1186/1753-6561-5-S9-S8

**Published:** 2011-11-29

**Authors:** Tesfaye M Baye, Hua He, Lili Ding, Brad G Kurowski, Xue Zhang, Lisa J Martin

**Affiliations:** 1 Division of Asthma Research, Cincinnati Children’s Hospital Medical Center, 3333 Burnet Avenue, Cincinnati, OH 45229, USA; 2 Department of Pediatrics, University of Cincinnati College of Medicine, 3333 Burnet Avenue, Cincinnati, OH 45229, USA; 3 Division of Biostatistics and Epidemiology, Cincinnati Children’s Hospital Medical Center, 3333 Burnet Avenue, Cincinnati, OH 45229, USA; 4 Division of Physical Medicine and Rehabilitation, Cincinnati Children’s Hospital Medical Center, 3333 Burnet Avenue, Cincinnati, OH 45229, USA; 5 Division of Human Genetics, Cincinnati Children’s Hospital Medical Center, 3333 Burnet Avenue, Cincinnati, OH 45229, USA

## Abstract

Next-generation sequencing technologies now make it possible to genotype and measure hundreds of thousands of rare genetic variations in individuals across the genome. Characterization of high-density genetic variation facilitates control of population genetic structure on a finer scale before large-scale genotyping in disease genetics studies. Population structure is a well-known, prevalent, and important factor in common variant genetic studies, but its relevance in rare variants is unclear. We perform an extensive population structure analysis using common and rare functional variants from the Genetic Analysis Workshop 17 mini-exome sequence. The analysis based on common functional variants required 388 principal components to account for 90% of the variation in population structure. However, an analysis based on rare variants required 532 significant principal components to account for similar levels of variation. Using rare variants, we detected fine-scale substructure beyond the population structure identified using common functional variants. Our results show that the level of population structure embedded in rare variant data is different from the level embedded in common variant data and that correcting for population structure is only as good as the level one wishes to correct.

## Background

With increasing availability of polymorphic molecular markers across genomes, examining population structure using a large number of loci has become a common practice in evolutionary biology and human genetics [1]. In assigning individual membership and inferences, investigators have found that some markers (or variants) are more informative than others [2]. In such cases, many loci are typed on samples from these populations, and subsets of these loci (typically those that appear most divergent between the populations) are chosen for analysis. Selecting and using only the most informative markers for population assignment can reduce both time and genotyping costs while retaining most of the power of the complete set of markers. However, currently more than 15 million common and rare single-nucleotide polymorphisms (SNPs) have been deposited in the 1000 Genomes Project database, and users of these data sets have several questions, including how many rare or common SNP loci are needed to get a good clustering or assignment and how much of the total variation is attributed to rare and common variants. In addition, the relationship between common and rare variants in terms of population structure remains unknown. To address this issue, we sought to answer the following two questions: Does a similar population structure (or inferred ancestry) exist in common and rare variants? From a population stratification perspective, how strongly are rare and common variants correlated? When both common and rare variants are obtained from the same participants, we are given the opportunity to investigate these questions directly. To answer these questions, we used rare and common SNPs from the Genetic Analysis Workshop 17 (GAW17) mini-exome sequence and ran a multivariate statistical analysis.

## Methods

For our analysis we used the data available from the 1000 Genomes Project as given in the GAW17 mini-exome sequence [3]. Seven of the 11 populations were included: Caucasians from the United States with northern and western European ancestry; Yoruba from Ibadan, Nigeria; Japanese from Tokyo; Han Chinese from Beijing; Chinese in metropolitan Denver, Colorado; Luhya in Webuye, Kenya; and Tuscans in Italy.

We first divided the data set into two groups: common functional variants and rare functional variants. Functional variants are variants that confer detectable (nonsynonymous) functional changes (both coding and regulatory) on the locus. Rare variants have a minor allele frequency (MAF) less than 5%, and variants with MAFs greater than (or equal to) 5% are common. In this study, the common functional variants consist of 1,379 SNPs and the rare functional variants consist of 12,193 SNPs. Both variants were summarized across the seven populations (697 samples). We used principal components analysis to reduce variable dimension, Structure analysis to assess ancestry, and discriminant analysis to predict population membership.

### Principal components analysis

Principal components analysis synthesizes information contained in a set of *n* observed variables (*M*_1_, …, *M_n_*) by seeking a new set of *k* (*k* <*n*) orthogonal variables (PC_1_, …, PC*_k_*), named PC; these variables are calculated from the eigen-decomposition of the covariance matrix *M*. The *j*th principal component (PC) is a linear combination of the observed variables:(1)

Where coefficients *α_ij_* are elements of the eigenvector corresponding to the *j*th eigenvalue. PCs were extracted in descending order from the corresponding eigenvalue that measures the variance of the original variables explained by each PC [4]. PCs were calculated using the R software (www.r-project.org). Because the axis of the PCs often correspond or co-segregated with geographic ancestries, we applied Structure analysis [5] to estimate the ancestry of each individual based on the seven populations. For each ancestry estimate, we performed 10,000 burn-in periods and 10,000 iterations. Separate analyses were performed for common and rare functional variants.

### Discriminant analysis

To avoid the limitation of a large number of SNPs compared to the relatively small number of individuals and the correlation occurring in allele frequencies, we ran a discriminant analysis using the uncorrelated top significant PCs. This analysis ensures that variables submitted to discriminant analysis are perfectly uncorrelated and that their number is less than that of analyzed individuals. For each data set (common and rare functional variants), we ranked markers based on the loading from the PCs eigenvector. From ranked markers, we selected the top subsets of markers (20–1,000 markers per subset) to evaluate population membership using prediction accuracy measures [4]. Prediction accuracy was calculated as the number of correctly classified individuals divided by the total number of individuals in the study.

## Results

### Population structure using common functional variants

**Figure 1:**
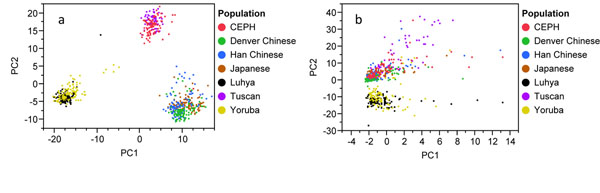
**Scatterplot of principal component axis one (PC1) and axis two (PC2) based on (a) common functional variants and (b) rare functional variants.** CEPH, European-descended population (U.S. Caucasians).

Principal components analysis using common functional variants revealed clear distinction among the three human geographic origins (Europe, Asia, and Africa) but not among the seven different populations studied (Figure 1). The first PC (which explains the largest portion of variation, 10.4%) distinguished between Africans and non-Africans, with samples from Yoruba and Han Chinese being widespread compared to the rest of the populations. The second PC, explainingÂ 6.6% of the total variation, distinguished between Europeans and non-Europeans. The analysis based on common variants required 388 PCs to account for 90% of the variation or population structure. Although the scree plots suggest that the first four PCs would be optimal to adjust for population stratification, we present the first two PCs in Figure 1 to demonstrate differences among the seven populations.

### Population structure using rare variants

**Figure 2:**
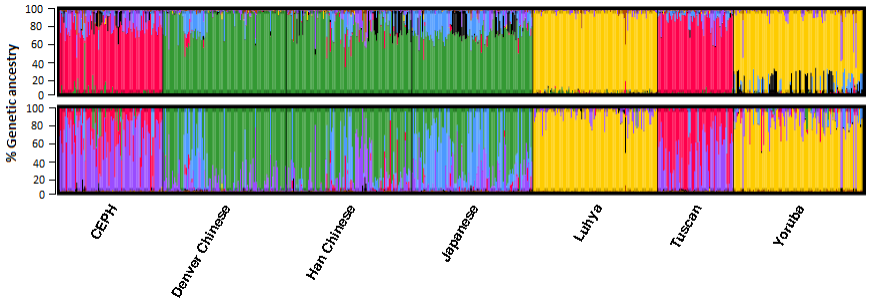
**Inferred genetic ancestry with even clusters from seven populations based on common (upper panel) and rare (lower panel) variants.** Each individual is represented by a thin vertical line, which is partitioned into seven colored segments that represent the individual’s estimated ancestry coefficients in the seven clusters. Individuals (separated by solid lines) are represented by bars on the *x*-axis, and ancestry proportion is given on the *y*-axis. The proportion of ancestry is illustrated by the amount of different color in each individual. CEPH, European-descended population (U.S. Caucasians).

Using PC1, we found that the populations were quite close to each other and did not show any clear clustering. Africans and non-Africans were distinguished only on the second PC (PC2) (Figure 1). The first PC contributed 1.6% and the second PC contributed 0.84% of the total variation among populations. The analysis based on rare variants required 532 significant PCs to account for 90% of the variation or population structure. The scree plots suggest that the first nine PCs should be included to adjust for population stratification; nonetheless, plotting PC1 against PC2 of the rare variants showed that most of these individuals had intermediate values between continental clusters of origin (Figure 1). Although individuals were classified according to geographic origin using principal components analysis, we observed substantial variability in the ancestral genetic background based on rare variants compared with common variants (Figure 2). Using rare variants, we identified an individual with primary European ancestry in a population sample of Yoruba.

### Population membership using discriminant analysis

**Figure 3:**
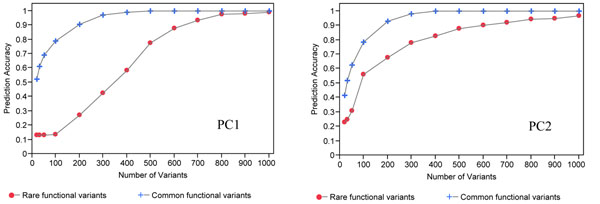
Predictive accuracy of common versus rare functional variants based on PC1 or PC2.

To assess how many markers are needed for accurate individual assignment to the correct population, we investigated the top subsets (20–1,000 markers per subset) of common or rare functional variants based on PC1- (or PC2-) selected SNPs (SNPs with the highest loading). Using common functional variants, 98% of individuals were assigned to their correct population using 400 SNPs (Figure 3). This was in contrast to the 1,000 rare functional variants needed to reach the same level of assignment of individuals to their correct ancestry.

### Distribution of MAF

**Figure 4:**
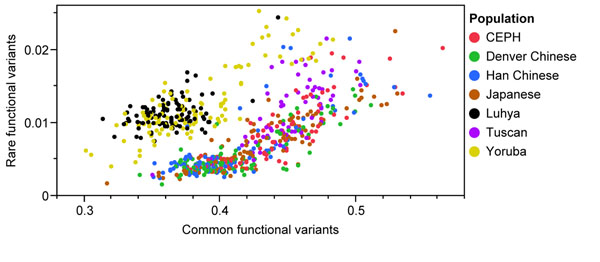
**Scatterplot of the 697 individuals using allele frequency for the common and rare variants.** CEPH, European-descended population (U.S. Caucasians).

European and Asian samples were more enriched in common functional variants, although African samples had more rare functional variants (Figure 4). It is also interesting to note that, although the actual variants contributing to population structure differed, we observed a modest statistical correlation (*r* = 0.38) between common and rare functional variants.

### Discussion and conclusions

Population structure is an important factor in genetic studies of common variants, but its relevance for rare variants is unclear. To our knowledge, the analysis presented here is the first population genetic structure study to explore rare versus common variants (using the same samples). To summarize genetic variation, we applied principal components analysis and demonstrated that the number of PCs required to account for population structure varied by the MAF of variants. Higher numbers of SNPs were required to account for a similar level of population structure when we used rare variants compared with common functional variants. In estimating ancestry proportion, using Structure analysis, we identified many Denver Chinese with more than 50% Japanese ancestry and many Tuscan individuals with more than 50% European ancestry. This result indicates the effectiveness of including rare variants to detect outliers even among geographically close populations. Also, a single individual with high (>90%) inferred European ancestry could be identified in the Yoruba population. However, this individual had less inferred European ancestry when we looked at common variants. This result further indicates the effectiveness of using rare variants to detect outliers among geographically close or distant populations.

Evolutionarily, many rare variants have occurred in recent human history; therefore they are expected to be population specific and to show greater population diversity than common variants [6,7]. Based on this hypothesis, one might expect rare functional variants to provide better predictive accuracy than common variants. Our result do not support this hypothesis, and using the same numbers of informative SNPs (such as 20), we found that the predictive accuracy for ancestral membership was 13% for rare variants and 52% for common variants. Thus fewer informative markers are required to assign individuals to their ancestral origin when we use common functional variants rather than rare functional variants. The confounding effect of high within-population diversity on allele frequencies in rare variants might have altered the results [8]. Thus it is critical to understand the population structure in a given sample set and to account for it before performing association analyses with other factors.

Our population classification using common functional variants performed similarly to studies using nonfunctional variants (data not shown), such that the first PC separated African populations and the second PC separated European-descent populations. Furthermore, within the African cluster there was more variability, which reflects the greater genetic diversity in samples of African origin [9]. Overall, the Luhya and Yoruba African samples, the U.S. and Tuscan European samples, and the Han Chinese, Denver Chinese, and Japanese Asian samples showed within-population clustering based on PC1 and PC2. These findings (common functional variants) appear to agree with Malécot’s isolation-by-distance model, which predicts that genetic similarity between populations will decrease exponentially as the geographic distance between them increases [10]. Examination of the isolation-by-distance model with rare functional variants showed that rare functional variants do not fit Malécot’s model; rather, they follow clinal trends as a result of the subtle signal of genetic diversity. Clines in allele frequencies may be the consequence of adaptation along an environmental gradient [11] or of genetic admixture occurring in secondary contact zones. Africans and U.S. Caucasians began to get close to 100% correct assignment when only 200 SNP loci were used, whereas Han Chinese and Japanese required 400 SNP loci. This is shown by the much shorter branch length for the Han Chinese/Japanese separation compared with the branch length of the U.S. Caucasian/Yoruba separation [12].

In summary, by restricting our analysis to each variant type independently instead of using global average estimates, we have reported for the first time that the optimal number of subpopulations is variant dependent. The variation in the number of PCs needed to account for population variation might indicate the detection of population structure that would have been missed if only common variants had been used. Thus correction for population structure is only as good as the type of variants chosen and the level of structure (finer or coarser) one wishes to correct. For example, if one wants to discriminate less differentiated groups, such as Denver Chinese from Han Chinese, one might need to pick additional markers that are known to exist in both populations but that vary in frequency. Future studies using the entire 1000 Genomes Project and other data sets will be needed to further explore how much of an estimate of ancestry is good enough to assign an individual to his or her founder population and to account for population structure as well as to confirm our findings.

## Competing interests

All authors declare no competing interest in relation to their work.

## Authors’ contributions

TMB conceived and wrote the manuscript. HH helped in statistical analysis. LJM, LD, XZ, and BGK helped with the writing of the manuscript. All authors read and approved the final manuscript.
